# Mortality Risk and Risk Factors in Patients with Posttraumatic Epilepsy: A Population-Based Cohort Study

**DOI:** 10.3390/ijerph16040589

**Published:** 2019-02-18

**Authors:** Wei-Jun Lin, Tomor Harnod, Cheng-Li Lin, Chia-Hung Kao

**Affiliations:** 1 Department of Emergency, China Medical University Hospital, Taichung 404, Taiwan; bbgun1208@gmail.com; 2 Department of Neurosurgery, Hualien Tzu Chi General Hospital, Buddhist Tzu Chi Medical Foundation, Hualien 970, Taiwan; tomorha@yahoo.com.tw; 3 College of Medicine, Tzu Chi University, Hualien 970, Taiwan; 4 Management Office for Health Data, China Medical University Hospital, Taichung 404, Taiwan; orangechengli@gmail.com; 5 College of Medicine, China Medical University, Taichung 404, Taiwan; 6 Graduate Institute of Biomedical Sciences and School of Medicine, College of Medicine, China Medical University, Taichung 404, Taiwan; 7 Department of Nuclear Medicine and PET Center, China Medical University Hospital, Taichung 404, Taiwan; 8 Department of Bioinformatics and Medical Engineering, Asia University, Taichung 413, Taiwan

**Keywords:** cohort study, mortality, National Health Insurance, posttraumatic epilepsy, traumatic brain injury

## Abstract

*Aim:* Use the National Health Insurance Research Database of Taiwan to determine whether patients with posttraumatic epilepsy (PTE) have an increased risk of mortality. *Methods:* Patients ≥20 years old ever admitted because of head injury (per International Classification of Diseases, Ninth Revision, Clinical Modification (ICD-9-CM) codes 850–854 and 959.01) during 2000–2012 were enrolled into a traumatic brain injury (TBI) cohort. The TBI cohort was divided into with PTE (ICD-9-CM code 345) and posttraumatic nonepilepsy (PTN) cohorts. We compared the PTE and PTN cohorts in terms of age, sex, and comorbidities. We calculated adjusted hazard ratios (aHRs) and 95% confidence intervals (CIs) of all-cause mortality risk in these cohorts. *Results:* Patients with PTE had a higher incidence rate (IR) of mortality than did patients with TBI alone (IR per 1000 person-years: 71.8 vs. 27.6), with an aHR 2.31 (95% CI = 1.96–2.73). Patients with PTE aged 20–49, 50–64, and ≥65 years had, respectively, 2.78, 4.14, and 2.48 times the mortality risk of the PTN cohort. Patients with any comorbidity and PTE had 2.71 times the mortality risk as patients in the PTN cohort. Furthermore, patients with PTE had 28.2 increased hospital days and 7.85 times as frequent medical visits per year compared with the PTN cohort. *Conclusion:* Taiwanese patients with PTE had approximately 2 times the mortality risk and an increased medical burden compared to patients with TBI only. Our findings provide crucial information for clinicians and the government to improve TBI outcomes.

## 1. Introduction

Traumatic brain injury (TBI) is common around the world, and it is associated with substantial early and long-term mortality, disabilities, costs, and subsequent development of various neurological deficits [1,2,3]. TBI generally reduces victims’ life expectancy by 6 years, regardless of age at the time of accident [4]. Moreover, patients surviving the early stage of TBI usually have higher risks of developing disabilities and comorbidities in their later lives, and this affects their life span. Baguley et al. reported that patients experiencing TBI had 5 to 10 times higher risks of dying from respiratory illnesses, other neurologic disorders, mental disorders, or digestive diseases [5].

Posttraumatic epilepsy (PTE) is a major type of symptomatic epilepsies those happen after various brain insults. It is a common comorbidity after TBI, and TBI is known as a leading cause of adulthood epilepsy [6,7]. The actual prevalence of PTE has been studied for decades and reported with wide-ranging figures from 1.3% to 53.3% [7,8,9], which might depend on the severity of the initial TBI. Possible latencies from the time of TBI to the development of PTE also vary greatly; this can be hours, days, or as remote as months or years after the initial injury [10,11]. In a prospective population-based study, a cohort of 792 patients had been followed for up to 14 years, and overall, the patients with various types of epilepsies had a 2.1-fold mortality risk than the general population [12]. Among them, patients with acute symptomatic epilepsy had a 3.0-fold mortality risk and patients with remote symptomatic epilepsy had a 3.7-fold mortality risk. Whether the PTE represents more damage from the brain insult to increase long-term mortality or the PTE itself would further increase the susceptibility of mortality after a TBI has still been unclear. 

However, the extent of increase in mortality risk and correlated risk factors for mortality in patients with PTE in Taiwan remain unknown, and the difference in mortality risk between patients with or without PTE should be investigated to elucidate future treatment strategies for TBI and PTE in Taiwan. We used a nationwide, population-based database to analyze and compare the mortality risks and risk factors between Taiwanese patients with PTE and those with TBI but without PTE. Because Taiwan is located in East Asia and Taiwanese people are similar to those of China and Southeast Asia [13], the findings of this study may aid future development and implementation of effective treatment strategies in other Asian countries. 

## 2. Methods

### 2.1. Data Source

The National Health Insurance Research Database (NHIRD) contains information regarding nearly all people in Taiwan. Implemented in 1995, the National Health Insurance (NHI) program offers thorough health care, including ambulatory and inpatient care, for nearly 99% of the 23.74 million people in Taiwan [14]. This study employed a retrospective cohort from the Longitudinal Health Insurance Database 2000 (LHID2000), a subset of the NHIRD, that consists of 1,000,000 people randomly selected from all those insured. Diseases were defined using the International Classification of Diseases, Ninth Revision, Clinical Modification (ICD-9-CM) codes. The Research Ethics Committee of China Medical University and Hospital in Taiwan approved of the research (CMUH104-REC2-115-CR3).

### 2.2. Patient Selection

The TBI cohort comprised LHID2000 insurants aged ≥20 years with head injury (ICD-9-CM 850–854 and 959.01) admitted during 2000–2012. For these patients, their head injury should not be mild and considered as moderate or severe head injury with TBI. The PTE cohort enrolled patients with diagnosis of epilepsy (ICD-9-CM codes 345) after TBI. The comparison cohort comprised patients without diagnosis of epilepsy after the TBI (posttraumatic nonepilepsy (PTN)). The date of first PTE diagnosis was defined as the index date, and the index date for the PTN group was a random month, day, and year after the head injury. Patients with head injury but without admission, and with diagnosis of epilepsy before the TBI admission, were excluded. We analyzed the distributions of the PTE and PTN cohorts in terms of age, sex, and comorbidities of alcohol-related illness (ICD-9-CM codes 291, 303, 305.00, 305.01, 305.02, 305.03, 571.0, 571.1, 571.3, 790.3, and V11.3), anxiety (ICD-9-CM code 300.00), mental disorders (ICD-9-CM codes 290–319), insomnia (ICD-9-CM codes 307.4 and 780.5), depression (ICD-9-CM codes 296.2, 296.3, 296.82, 300.4, and 311), stroke (ICD-9-CM codes 430–438), chronic obstructive pulmonary disease (COPD) (ICD-9-CM codes 491, 492, and 496), coronary artery disease (CAD) (ICD-9-CM code 410–414), diabetes mellitus (ICD-9-CM code 250), hypertension (ICD-9-CM code 401–405), and hyperlipidemia (ICD-9-CM code 272). We analyzed adjusted hazard ratios (aHRs) and 95% confidence intervals (CIs) of all-cause mortality risk in these cohorts using 2 matching methods. One method was frequency matching based on age and sex, and the other was propensity-score matching based on age, sex, and comorbidity. Both methods matched in a 1:1 manner.

### 2.3. Statistical Analysis

We applied the Student *t* test for mean age and chi-square test for age, sex, and strata of comorbidity. We defined incidence as the number of events divided by person-years and derived the crude HRs and aHRs based on multivariable Cox proportional hazard regression model after adjusting for age, sex, and comorbidities. Through stepwise regression analyses, we analyzed the effects of factors of age, stroke, diabetes, COPD, anxiety, depression, and sex (male vs. female) on average hospital days per year (all-cause admission) between PTE and PTN as well as the effects of age, hyperlipidemia, CAD, insomnia, mental disorders, hypertension, COPD, diabetes, and depression on the frequency of medical visits per year under propensity-score matching between the cohorts. Moreover, we derived the cumulative incidences of mortality in the PTE and PTN cohorts with the Kaplan-Meier method and detected differences through log-rank testing. All analyses were conducted using SAS 9.4 software (SAS Institute, Cary, NC, USA). A *p* value of <0.05 was considered statistically significant.

## 3. Results

### 3.1. Demographics of Study Cohorts

For frequency matching analysis, the sizes of the PTE and PTN cohorts were both 1425 patients. The distributions differed in alcohol-related illness, anxiety, mental disorders, insomnia, depression, COPD, CAD, diabetes, and hypertension (all *p* < 0.001) (Table 1). For propensity-score matching, the sizes of the PTE and PTN cohorts were both 891 patients, and the distributions of the cohorts were similar in age, sex, and comorbidity (Table 1).

### 3.2. Mortality Risk in Patients with PTE

**Figure 1:**
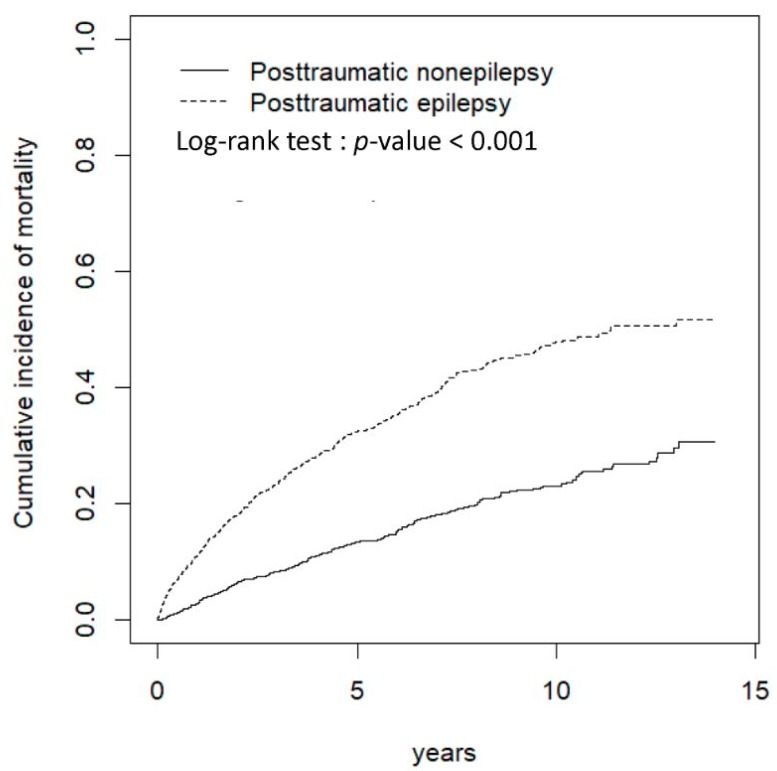
Cumulative incidence of mortality between patients with and without posttraumatic epilepsy after traumatic brain injury.

The cumulative incidences of mortality in the PTE and PTN cohorts significantly differed (log-rank test: *p* < 0.001) (Figure 1). In frequency matching, patients with PTE were 2.53 times as susceptible to mortality as those in the PTN cohort (incidence rate (IR) per 1000 person-years: 71.8 vs. 27.6), with an aHR of 2.31 (95% CI = 1.96–2.73). For propensity-score matching, the PTE cohort had 1.96 times the risk of mortality of the PTN cohort (IR: 61.3 vs. 30.8), with an aHR of 2.28 (95% CI = 1.88–2.75) (Table 2).

In frequency matching, the patients with PTE aged 20–49, 50–64, and ≥65 years had, respectively, 2.78, 4.14, and 2.48 times the risk of mortality as the PTN cohort, with aHRs of 1.78, 3.39, and 2.00 (Table 3). Women and men with PTE had, respectively, 2.74 and 2.44 times the mortality risk of those in the PTN cohort (IR for women, 74.9 vs. 26.6; for men, 70.6 vs. 28.0), with aHRs of 2.54 and 2.21. When considering the effects of comorbidity, patients with PTE and any one of the comorbidities had 2.15 times the risk of mortality as patients in the PTN cohort (IR, 78.9 vs. 36.2), with an aHR of 2.71. However, patients with PTE without any comorbidity still had 1.98 times the risk of mortality of the PTN cohort after adjusting for other covariates. In further analysis through propensity-score matching, patients with PTE aged 20–49, 50–64, and ≥65 years had, respectively, 2.10, 2.41, and 2.05 times the mortality risk of patients in the PTN cohort (IR for 20–49 years, 33.8 vs. 16.0; 50–64 years, 107.1 vs. 43.7; ≥65 years, 203.0 vs. 99.6), with aHRs of 2.13, 2.75, and 2.17. Analysis of sex revealed that women and men with PTE had, respectively, 2.11 and 1.89 times the mortality risk than their counterparts in the PTN cohort (IR for women, 66.0 vs. 31.0; men, 59.3 vs. 30.8), with aHRs of 2.42 and 2.24. Patients with PTE and any one of the comorbidities had 1.92 times the risk of mortality of the PTE cohort (IR, 70.7 vs. 36.4), with an aHR of 2.20 (Table 3).

### 3.3. AED Use and Mortality Risk

Antiepileptic drug (AED) would be usually given for PTE prevention and treatment. We further analyzed the association of the number of AED use and the mortality risk of patients. Among the 1425 patients with PTE, 42 (2.95%) did not use AED, 219 (15.37%) had 1 AED, 515 (36.14%) had 2 AEDs, and 649 (45.54) had >2 AED after their TBIs. Compared to the PTN cohort, the patients with PTE and no use of AED, with 1 AED, 2 AEDs, and >2 AEDs had respectively, 0.83, 2.05, 2.28, and 2.63 times the risk of mortality. When compared to the patients with PTE and no use of AED, the patients who used 1 AED, 2 AEDs, and >2 AEDs had 2.48, 2.89, and 3.30 times the risk of mortality respectively (Table 4). 

### 3.4. Increased Medical Burden in Patients with PTE

Through propensity-score matching, patients with PTE had averaged 28.2 hospital days increased per year than patients in the PTE cohort. The average hospital days per year increased by 0.51 day when age increased by 1 year. Stroke, diabetes, COPD, and depression resulted in, respectively, 12.8, 11.9, 10.2, and 19.1 more average hospital days per year. Anxiety resulted in 13.2 fewer average hospital days per year. We noted that male patients averaged 6.19 more hospital days per year than female patients (Table 5).

Under propensity-score matching, patients with PTE had 7.85 times as many medical visits per year than did patients in the PTN cohort. The frequency of medical visits per year increased by 0.2 visits when age increased by 1 year. Hyperlipidemia, CAD, insomnia, mental disorders, hypertension, COPD, and diabetes, respectively, led to 5.82, 5.95, 5.68, 4.15, 4.73, 5.17, and 5.38 times the frequency of medical visits (Table 6).

## 4. Discussion 

Taiwanese patients with TBI and PTE had more than twice the mortality risk of those with TBI and without PTE. Overall, in men with TBI, the ones with and without PTE had mortality rates of 70.6 per 1000 person-years and 28.0 per 1000 person-years, respectively; in women with TBI, the ones with and without PTE had mortality rates of 74.9 per 1000 person-years and 26.6 per 1000 person-years, respectively. The existing comorbidities of alcohol-related illness, anxiety, mental disorders, insomnia, depression, stroke, COPD, CAD, diabetes, hypertension, and hyperlipidemia during the post-TBI follow-up also led to a significant increase in subsequent mortality among patients with PTE. In previous studies, the highest mortality risk has been observed in old-aged patients with epilepsy [15,16]. As a symptomatic epilepsy, PTE mortality risk in this study was notably higher in patients aged 50–64 years than in those older than 64 years. Our study revealed some notable differences compared to similar studies previously conducted in Western countries where mortality risk in adults with epilepsy was strongly affected by increasing age. Such findings suggest reconsidering the roles of age and post-TBI comorbidities for long-term mortality when the patients develop PTE after TBI. By our results, PTE, alcohol-related illness, anxiety, mental disorders, insomnia, depression, stroke, COPD, CAD, diabetes, hypertension, and hyperlipidemia in post-TBI patients should be early and aggressively treated to increase their life span and reduce medical burden, especially in middle-aged patients with 50–64 years old and PTE. Moreover, we observed that 81.68% of patients with PTE had 2 or more AEDs and associated with a trend of higher mortality risk than the ones had none or 1 AED. The controversial benefit from polytherapy of AED use in patients with PTE was demonstrated by our analysis. The future indication of AED use for PTE should be carefully reconsidered and studied more to improve clinical practice. 

Epileptogenesis involves pathological alterations at the molecular and cellular levels. It can increase brain excitability and eventually enable brain tissue to generate spontaneous recurrent seizures after TBI. Because we cannot successfully determine which one will develop epileptogenesis and subsequent PTE in current practice [17,18], we seek to understand the incidence and risk factors for PTE and try to reduce the related mortality risk instead of trying to identify epileptogenesis directly. Among the various causes of mortality in patients with epilepsy, deaths directly caused by epileptic seizures, such as sudden unexpected death from epilepsy, status epilepticus, burns, drowning, and cervical fracture after seizure, are uncommon [12,19]. The leading causes of death in patients with epilepsy are noncerebral neoplasm, cardiovascular, and cerebrovascular disease, accounting for 59% of deaths [19]. Our study was limited by the inability to confirm the cause of each individual’s death from the NHIRD. However, we identified PTE, stroke, diabetes, COPD, and depression as the leading factors increasing hospital day in patients after TBI. Moreover, PTE, CAD, diabetes, COPD, and depression are the leading factors of more frequent medical visits in patients after TBI. As we know, irritability, agitation, psychosis, and suicidal thoughts are all potential risk factors of mortality in patients with TBI and those with PTE. The relationships of hospital days, frequency of medical visits, PTE, and mortality suggest that some existing comorbid disorders interact with PTE to increase the subsequent mortality and medical burden in these patients. Due to the fact that we already controlled the psychobehavior comorbidities, such as alcohol-related illness, anxiety, mental disorders, insomnia, and depression in the analysis, we suggest that PTE could be considered as an independent predisposing factor for post-TBI mortality and poor outcome in Taiwan, irrespective of comorbidities. Additional studies in different countries are required to confirm whether this result is applicable elsewhere.

In this study, we analyzed inpatient cases with both first-time TBI and possible repeated TBI events to estimate the correlation of PTE and subsequent all-cause mortality risk. The large study population, being fairly unselected and only excluding the mild head injury cases treated at outpatient service, provided the main strength of this study. Patients with PTE would be much less likely to return to ordinary lives than those do not develop PTE after TBIs. A recent study depicted that various accidents and cerebrovascular diseases were more common in patients with PTE, whereas post-TBI dementia, suicide, and violent behavior were more common in patients with PTN. Alcohol-related diseases, accidents, suicide, and violence behavior were more prevalent specifically in young patients with PTE [20]. All these would be the potential causes of long-term mortality in those with PTE. Moreover, we intentionally excluded the deaths during TBI-related admission because severe TBI itself might directly lead to death and that would be not related to PTE. All the analyses were based on death after discharge from TBI-related admission, and all deaths were reported and analyzed using inpatient data for high validity in this study. The NHI universal health care system guarantees the residents of Taiwan equal access to medical service throughout the country with few disparities in access to inpatient services and outcomes between different hospitals and areas in Taiwan [21,22]. The proportion of patients with TBI or PTE dying outside hospitals is low under the conditions of our study, with a rare possibility of underestimation bias in the results. 

However, this study had other limitations. First, we could not directly contact the patients because their identities were anonymized in the NHIRD. Therefore, the study design did not include details regarding the severity and actual type of their TBIs by ICD code, whether a patient received any neurosurgical intervention regarding to the TBI at first admission, or severity of their PTE, or how PTE was treated individually. Some AED might be given as PTE prevention in the patients actually without PTE. All of these might affect the individual outcome and as confounding factors in this study. Second, although the NHI program performs thorough quarterly reviews to ensure that the files are accurate and false claims are heavily penalized, rare occurrences of miscoding may have nevertheless occurred in the NHIRD. Finally, although our study design included adequate controls for numerous confounding factors, unmeasured or unknown confounders, such as family or social support for PTE and PTN patients, can mediate mortality and morbidity to generate bias. However, after considering the aforementioned limitations, our results indicated that the sample size was sufficient to statistically demonstrate the increased subsequent mortality risk in patients with PTE.

## 5. Conclusions

Taiwanese patients with TBI and PTE had approximately twice the mortality risk as those with TBI without PTE, and the highest risk was noted in patients aged 50–64 years. Our findings provide vital information for clinicians and the government to improve the survival and alleviate the medical burden of patients with TBI in the future. Additional studies in different countries are required to confirm whether this result is globally applicable.

## Figures and Tables

**Table 1:** Distribution of age, sex, and comorbidity in PTE (posttraumatic epilepsy) and PTN (posttraumatic nonepilepsy) cohorts with and without propensity-score matching.

Variable						Propensity Score Matched				
	Posttraumatic Nonepilepsy *n* = 1425		Posttraumatic Epilepsy *n* = 1425			Posttraumatic Nonepilepsy *n* = 891		Posttraumatic Epilepsy *n* = 891		
	*n*	%	*n*	%	*p*-Value	*n*	%	*n*	%	*p*-Value
Age, year					0.99					0.98
20–49	623	43.7	623	43.7		583	65.4	582	65.3	
50–64	321	22.5	321	22.5		135	15.2	133	14.9	
65+	481	33.8	481	33.8		173	19.4	176	19.8	
Mean (SD) ^§^	54.2	19.7	54.5	19.6	0.74	54.6	19.9	54.4	19.9	0.84
Sex					0.99					0.92
Female	435	30.5	435	30.5		290	32.6	288	32.3	
Male	990	69.5	990	69.5		601	67.5	603	67.7	
Comorbidity										
Alcohol-related illness	124	8.70	300	21.1	<0.001	114	12.8	102	11.5	0.38
Anxiety	278	19.5	422	29.6	<0.001	226	25.4	229	25.7	0.87
Mental disorders	547	38.4	890	62.5	<0.001	448	50.3	443	49.7	0.81
Insomnia	722	50.7	1079	75.7	<0.001	591	66.3	587	65.9	0.84
Depression	98	6.88	200	14.0	<0.001	89	9.99	89	9.99	0.99
Stroke	136	9.54	393	27.6	0.06	127	14.3	133	14.9	0.69
Chronic obstructive pulmonary disease (COPD)	211	14.8	377	26.5	<0.001	178	20.0	188	21.1	0.56
Coronary artery disease (CAD)	304	21.3	346	24.3	<0.001	220	24.7	214	24.0	0.74
Diabetes	153	10.7	244	17.1	<0.001	121	13.6	126	14.1	0.73
Hypertension	537	37.7	705	49.5	<0.001	389	43.7	381	42.8	0.70
Hyperlipidemia	307	21.5	328	23.0	0.34	200	22.5	201	22.6	0.95

Chi-square test; ^§^ *t* test.

**Table 2:** Overall incidence (per 1000 person-years) and hazard ratio of mortality with and without propensity-score matching between the PTE and PTN cohorts.

Variable			Propensity Score Matched	
	Posttraumatic Nonepilepsy	Posttraumatic Epilepsy	Posttraumatic Nonepilepsy	Posttraumatic Epilepsy
	(*n* = 1425)	(*n* = 1425)	(*n* = 891)	(*n* = 891)
Mortality				
Person-years	9126	7438	5577	4846
Follow-up time (y)	6.40 ± 3.80	5.22 ± 3.79	4.36 ± 3.17	3.91 ± 2.97
No. of event	252	534	172	297
Incidence rate	27.6	71.8	30.8	61.3
Crude HR (95% CI)	1 (Reference)	2.53 (2.18, 2.94) ***	1 (Reference)	1.96 (1.62, 2.36) ***
Adjusted HR ^a^ (95% CI)	1 (Reference)	2.31 (1.96, 2.73) ***	1 (Reference)	2.28 (1.88, 2.75) ***

^a^ Adjusted for age, sex, alcohol-related illness, anxiety, mental disorders, insomnia, depression, stroke, COPD, CAD, diabetes, hypertension, and hyperlipidemia; *** *p* < 0.001.

**Table 3:** Incidence and hazard ratio for mortality stratified by age, sex, and comorbidity of patients with and without PTE after TBI (traumatic brain injury).

Variable							Propensity Score Matched					
	Posttraumatic Nonepilepsy		Posttraumatic Epilepsy				Posttraumatic Nonepilepsy		Posttraumatic Epilepsy			
	*n* = 1425		*n* = 1425				*n* = 891		*n* = 891			
	Event No.	Rate	Event No.	Rate	Crude HR (95% CI)	Adjusted HR ^a^ (95% CI)	Event No.	Rate	Event No.	Rate	Crude HR (95% CI)	Adjusted HR ^a^ (95% CI)
Age, year												
20–49	53	11.5	133	32.3	2.78 (2.02, 3.83) ***	1.78 (1.23, 2.56) **	65	16.0	126	33.8	2.10 (1.56, 2.83) ***	2.13 (1.57, 2.89) ***
50–64	34	16.1	114	68.7	4.14 (2.82, 6.07) ***	3.39 (2.25, 5.11) ***	34	43.7	63	107.1	2.41 (1.59, 3.67) ***	2.75 (1.74, 4.34) ***
65+	165	68.6	287	172.5	2.48 (2.04, 3.00) ***	2.00 (1.62, 2.45) ***	73	99.6	108	203.0	2.05 (1.51, 2.77) ***	2.17 (1.59, 2.98) ***
Sex												
Female	71	26.6	160	74.9	2.74 (2.07, 3.63) ***	2.54 (1.88, 3.44) ***	53	31.0	96	66.0	2.11 (1.51, 2.94) ***	2.42 (1.72, 3.42) ***
Male	181	28.0	374	70.6	2.44 (2.05, 2.92) ***	2.21 (1.82, 2.69) ***	119	30.8	201	59.3	1.89 (1.51, 2.37) ***	2.24 (1.78, 2.82) ***
Comorbidity												
None	41	12.5	18	20.0	1.59 (0.92, 2.78)	1.98 (1.12, 3.47) *	15	11.9	18	20.0	1.64 (0.83, 3.26)	1.67 (0.83, 3.33)
With any one	211	36.2	516	78.9	2.15 (1.83, 2.53) ***	2.71 (2.31, 3.19) ***	157	36.4	279	70.7	1.92 (1.58, 2.34) ***	2.20 (1.81, 2.68) ***

Rate: per 1000 person-years; HR: relative hazard ratio; ^a^ Adjusted for age, sex, alcohol-related illness, anxiety, mental disorders, insomnia, depression, stroke, COPD, CAD, diabetes, hypertension, and hyperlipidemia; * *p* < 0.05, ** *p* < 0.01, *** *p* < 0.001.

**Table 4:** Incidence and hazard ratio of mortality compared among PTE patients with different number of AED (anti-epileptic drug) use and compared to PTN.

Variables	*n*	Event	Person-Years	Rate	Crude HR (95% CI)	Adjusted HR ^a^ (95% CI)	Crude HR (95% CI)	Adjusted HR ^a^ (95% CI)
Posttraumatic nonepilepsy	1425	252	9126	27.6	1(Reference)	1(Reference)		
Posttraumatic epilepsy								
Without antiepileptic drug	42	5	289	17.3	0.63 (0.26, 1.55)	0.83 (0.34, 2.01)	1 (Reference)	1 (Reference)
One antiepileptic drug	219	68	1433	47.4	1.73 (1.32, 2.26) ***	2.05 (1.56, 2.69) ***	2.70 (1.09, 6.69) *	2.48 (0.99, 6.18)
Two antiepileptic drugs	515	198	2846	69.6	2.48 (2.06, 2.99) ***	2.28 (1.87, 2.78) ***	3.85 (1.58, 9.34) **	2.89 (1.18, 7.07) *
More than two antiepileptic drugs	649	263	2870	91.6	3.13 (2.63, 3.72) ***	2.63 (2.17, 3.19) ***	4.78 (1.97, 11.6) ***	3.30 (1.35, 8.08) **

Rate: Per 1000 person-years; HR: Relative hazard ratio; ^a^ Adjusted for age, sex, alcohol-related illness, anxiety, mental disorders, insomnia, depression, stroke, COPD, CAD, diabetes, hypertension, and hyperlipidemia; * *p* < 0.05, ** *p* < 0.01, *** *p* < 0.001. Antiepilepsy drug including potassium clorazepate, phenobarbital, diazepam, zonisamide, valproic acid, topiramate, tiagabine, pregabalin, oxcarbazepine, levetiracetam, lamotrigine, gabapentin, carbamazepine, phenytoin, clonazepam, and primidone.

**Table 5:** Stepwise regression analysis for average hospital days per year (all-cause admission) through propensity-score matching.

Variable	Parameter Estimate	Standard Error	95% CI
Intercept	−27.9	5.28	(−38.2, −17.5) ***
Posttraumatic epilepsy vs. Posttraumatic nonepilepsy	28.2	2.72	(22.9, 33.5) ***
Age	0.51	0.08	(0.35, 0.68) ***
Stroke	12.8	4.19	(4.57, 21.0) **
Diabetes	11.9	4.13	(3.74, 20.0) **
COPD	10.2	3.79	(2.78, 17.6) **
Anxiety	−13.2	3.65	(−20.4, −6.07) ***
Depression	19.1	5.17	(8.94, 29.2) ***
Sex (male vs. female)	6.19	3.02	(0.26, 12.1) *

* *p* < 0.05, ** *p* < 0.01, *** *p* < 0.001.

**Table 6:** Stepwise regression analysis for frequency of medical visits per year after propensity-score matching.

Variable	Parameter Estimate	Standard Error	95% CI
Intercept	2.44	2.22	(−1.91, 6.89)
Posttraumatic epilepsy vs. Posttraumatic nonepilepsy	7.85	1.27	(5.36, 10.3) ***
Age	0.20	0.05	(0.11, 0.29) ***
Hyperlipidemia	5.82	1.75	(2.38, 9.26) ***
CAD	5.95	1.85	(2.33, 9.58) ***
Insomnia	5.68	1.46	(2.81, 8.55) ***
Mental disorders	4.15	1.43	(1.35, 6.95) **
Hypertension	4.73	1.81	(1.18, 8.28) **
COPD	5.17	1.78	(1.67, 8.68) **
Diabetes	5.38	2.01	(1.44, 9.32) **
Depression	5.06	2.26	(0.63, 9.50)

** *p* < 0.01, *** *p* < 0.001.

## Abbreviations

aHR	adjusted hazard ratio
CI	confidence interval
NHIRD	National Health Insurance Research Database
ICD-9-CM	International Classification of Diseases, Ninth Revision, Clinical Modification

## References

[1] Centers for Disease Control and Prevention (2015). Report to Congress. Traumatic brain injury in the United States: Epidemiology and rehabilitation. Atlanta: National Center for Injury Prevention and Control, Division of Unintentional Injury Prevention. https://www.cdc.gov/traumaticbraininjury/pubs/congress_epi_rehab.html.

[2] Perry D.C., Sturm V.E., Peterson M.J., Pieper C.F., Bullock T., Boeve B.F., Miller B.L., Guskiewicz K.M., Berger M.S., Kramer J.H. (2016). Association of traumatic brain injury with subsequent neurological and psychiatric disease: A meta-analysis. J. Neurosurg..

[3] Institute of Medicine (2009). Gulf war and health. Volume 7: Long-Term Consequences of Traumatic Brain Injury.

[4] Ventura T., Harrison-Felix C., Carlson N., Diguiseppi C., Gabella B., Brown A., Devivo M., Whiteneck G. (2010). Mortality after discharge from acute care hospitalization with traumatic brain injury: A population-based study. Arch. Phys. Med. Rehabil..

[5] Baguley I.J., Nott M.T., Howle A.A., Simpson G.K., Browne S., King A.C., Cotter R.E., Hodgkinson A. (2012). Late mortality after severe traumatic brain injury in New South Wales: A multicentre study. Med. J. Aust..

[6] Hesdorffer D.C., Logroscino G., Benn E.K., Katri N., Cascino G., Hauser W.A. (2011). Estimating risk for developing epilepsy: A population-based study in Rochester, Minnesota. Neurology.

[7] Annegers J.F., Hauser W.A., Coan S.P., Rocca W.A. (1998). A population-based study of seizures after traumatic brain injuries. N. Engl. J..

[8] Xu T., Yu X., Ou S., Liu X., Yuan J., Huang H., Yang J., He L., Chen Y. (2017). Risk factors for posttraumatic epilepsy: A systematic review and meta-analysis. Epilepsy Behav..

[9] Christensen J., Pedersen M.G., Pedersen C.B., Sidenius P., Olsen J., Vestergaard M. (2009). Long-term risk of epilepsy after traumatic brain injury in children and young adults: A population-based cohort study. Lancet.

[10] Rao V., Parko K. (2015). Clinical approach to posttraumatic epilepsy. Semin. Neurol..

[11] Mahler B., Carlsson S., Andersson T., Adelöw C., Ahlbom A., Tomson T. (2015). Unprovoked seizures after traumatic brain injury: A population-based case-control study. Epilepsia.

[12] Lhatoo S.D., Johnson A.L., Goodridge D.M., MacDonald B.K., Sander J.W., Shorvon S.D. (2001). Mortality in epilepsy in the first 11 to 14 years after diagnosis: Multivariate analysis of a long-term, prospective, population-based cohort. Ann. Neurol..

[13] Ying Y.W., Han M. (2008). Cultural orientation in Southeast Asian American young adults. Cultur. Divers. Ethn. Minor. Psychol..

[14] National Health Insurance Research Database. http://nhird.nhri.org.tw/en/index.html.

[15] Forsgren L., Hauser W.A., Olafsson E., Sander J.W., Sillanpää M., Tomson T. (2005). Mortality of epilepsy in developed countries: A review. Epilepsia.

[16] Levira F., Thurman D.J., Sander J.W., Hauser W.A., Hesdorffer D.C., Masanja H., Odermatt P., Logroscino G., Newton C.R. (2017). Epidemiology Commission of the International League Against Epilepsy. Premature mortality of epilepsy in low- and middle-income countries: A systematic review from the Mortality Task Force of the International League Against Epilepsy. Epilepsia.

[17] Lusardi T.A., Akula K.K., Coffman S.Q., Ruskin D.N., Masino S.A., Boison D. (2015). Ketogenic diet prevents epileptogenesis and disease progression in adult mice and rats. Neuropharmacology.

[18] Pitkänen A., Kharatishvili I., Karhunen H., Lukasiuk K., Immonen R., Nairismägi J., Gröhn O., Nissinen J. (2017). Epileptogenesis in experimental models. Epilepsia.

[19] Keezer M.R., Bell G.S., Neligan A., Novy J., Sander J.W. (2016). Cause of death and predictors of mortality in a community-based cohort of people with epilepsy. Neurology.

[20] Uski J., Lamusuo S., Teperi S., Löyttyniemi E., Tenovuo O. (2018). Mortality after traumatic brain injury and the effect of posttraumatic epilepsy. Neurology.

[21] Huang N., Yip W., Chang H.J., Chou Y.J. (2006). Trends in rural and urban differentials in incidence rates for ruptured appendicitis under the National Health Insurance in Taiwan. Public Health.

[22] Shu C.C., Lin J.W., Lin Y.F., Hsu N.C., Ko W.J. (2011). Evaluating the performance of a hospitalist system in Taiwan: A pioneer study for nationwide health insurance in Asia. J. Hosp. Med..

